# Research on aerobic fitness in children and adolescents: a bibliometric analysis based on the 100 most-cited articles

**DOI:** 10.3389/fmed.2024.1409532

**Published:** 2024-09-25

**Authors:** Rui Xia, Liu Yang, Chaomei Liang, Dongye Lyu, Wanli Zang, Guanrong Sun, Jin Yan

**Affiliations:** ^1^School of Physical Education, Chaohu University, Hefei, China; ^2^School of Education, College of Human and Social Futures, University of Newcastle, Newcastle, NSW, Australia; ^3^Library of Beijing Sport University, Beijing, China; ^4^College of Education Sciences, The Hong Kong University of Science and Technology, Guangzhou, Guangzhou, China; ^5^Postgraduate School, University of Harbin Sport, Harbin, China; ^6^Department of Public Physical and Art Education, Zhejiang University, Hangzhou, China; ^7^School of Physical Education and Sports Science, Soochow University, Suzhou, China

**Keywords:** cardiorespiratory fitness, child, adolescent, bibliometric analysis, VOS viewer, visualization

## Abstract

**Introduction:**

This study aims to conduct a bibliometric analysis of the 100 most-cited articles to examine research trends, hot topics, and gaps in aerobic fitness research in children and adolescents, addressing the lack of evidence synthesis.

**Methods:**

The Web of Science Core Collection database was used for literature search, and bibliometric characteristics of the included research articles were imported and calculated. Descriptive statistics and visualizations by the VOS viewer were used for the presentation of bibliometric characteristics.

**Results:**

The 100 most cited articles received an average of 104 citations. British Journal of Sports Medicine and Medicine and Science in Sports and Exercise were the two top journals that published aerobic fitness research in children and adolescents. The United States was the top country that contributed to the most-cited research articles. Three top research topics were identified from the analysis, such as neuroscience, developmental psychology, and aerobic health.

**Conclusion:**

Aerobic fitness research in children and adolescents has received much attention and interest since 2000. The most contributing authors in this research field were from developed countries, such as the United States, and cognition and health-related research were priorities.

## 1 Introduction

Aerobic fitness, or cardiovascular fitness, is the ability of the heart, lungs, and blood vessels to supply oxygen to muscles during sustained physical activity. It is essential for overall health and endurance, allowing the body to perform oxygen-demanding activities over extended periods (1, 2). Many vital and consistent conclusions have been drawn from the past series of published studies on the importance of aerobic fitness (3–7). This literature shows that aerobic fitness is essential to physical and mental health. Hendrikse et al. (8) conducted a study on the effects of regular aerobic exercise in which they evaluated 40 healthy young to middle-aged adults in two groups. It was found that the group that performed a higher level of training was effective in improving mental health and improving conditions such as depression and schizophrenia. Cheng and Guan (9) have also obtained similar results, stating that aerobic exercise can soothe the body and mind of adults, reduce life stress and anxiety, and improve mental health. According to Chaddock-Heyman et al. (10), aerobic fitness is strongly linked to how efficiently people’s brains function. In another study conducted on primary school students aged 7–10 who were taken as the research objects, they found that the students surveyed achieved higher levels of aerobic fitness, which would benefit the building of these students’ brain structures and accelerate their improvement in brain function (10). Another point is that young people’s mental health has always been a social concern. When adolescents have problems such as depression, anxiety, irritability, and even violent tendencies, aerobic fitness can effectively reduce the risk of mental health and poor mental health (11). Specifically, aerobic fitness increases the efficiency of synaptic transmission of monoamines, which include the three major neurotransmitters: norepinephrine, dopamine, and serotonin (12). These can improve people’s psychological conditions very well, and some antidepressants also draw on this principle (13). Since the outbreak of COVID-19, the physical and mental disabilities people face have become more prominent. The study by Johnson et al. (14) brought some good news. People with a low mood can experience an improvement through aerobic fitness. This improvement will extend to affect the individual’s physical function so that the individual body will produce more plasticity and positive response. Therefore, aerobic fitness plays a vital role in both middle-aged people and teenagers, whether to maintain mental health or promote physical health.

Research on aerobic fitness in children and adolescents can be divided into primary dimensions, including surveillance and monitoring, measurement and evaluation, correlation, and determinant and health impacts of aerobic fitness (15–17). These research areas can answer different research questions to highlight the health significance of aerobic fitness in children and adolescents (18), advocate for health policy initiatives, and implement efficient health strategies for health promotion (18). Despite the meaning of each research area, those studies can address limited research gaps, whereas these studies cannot provide a comprehensive landscape of research status. A better understanding of the current research status of aerobic fitness in children and adolescents helps seek current research hotspots and direct future research (19, 20). Using bibliometrics analysis to form the research landscape on aerobic fitness may be a suitable and feasible analytical approach (21).

Bibliometrics was first proposed by Alan Pritchard in 1969 (22). Bibliometrics is the application of mathematics and statistics to calculate and analyze different aspects of written information to reveal data processing or to study the nature and trends of development in a subject (23). Bibliometrics research is a technique that can provide quantitative analysis (24). In recent years, bibliometrics has gradually changed from theory to practical application, which makes the research structure of theory, method, and application more complete (25). In terms of application, due to the lack of unified database resources, it is difficult for researchers to collect data, which requires knowledge of probability statistics and decision theory (26).

However, as far as we know, few studies have touched upon the characteristics, development, and growth of the relevant subject. Therefore, the study is conducted to perform a bibliometric analysis of aerobic fitness among adolescents and children. A comprehensive perspective can be provided to probe into the problem, identify weak or promising areas, and determine the collaboration patterns. Therefore, policymakers and administrators can evaluate research performance on aerobic fitness among adolescents and children and make evidence-based decisions, while educational professionals and scholars can assess their domain and find inspiration for their future works.

## 2 Materials and methods

### 2.1 Study design and search strategy

The methodology was guided by a prior bibliometric analysis of the most-cited publications on physical activity (27, 28). For our literature search, we opted for the Web of Science Core Collection (including Science Citation Index Expanded, Social Sciences Citation Index, Arts and Humanities Citation Index, and Emerging Sources Citation Index) database, which contains more than thousands of peer-reviewed, high-quality publications in the field of more than 250 medical, social science, and humanities areas throughout the world. In addition, the database identifies the authors, countries, and keywords associated with each publication, which was essential for the analysis of this study (29). Our literature search was completed at the end of March 2024. Ethical approval was exempted for this research since it did not include any human participants or animal models in any way.

The following combinations of terms were used to design the search strategy: TI = [(child* OR adolescent* OR youth OR teen* OR student* OR girl* OR boy* OR kid*) NOT (university OR college OR adult*)] AND TI = (“aerobic fitness” OR “cardiorespiratory fitness”).

### 2.2 Publication selection and data extraction

1,117 publications were obtained from the Web of Science Core Collection database. The inclusion criteria were: (1) the main variable of interest in the study was aerobic fitness (or cardiorespiratory fitness), (2) the study population was children and/or adolescents, (3) the type of article was “articles or reviews” and, (4) the language was limited to “English.”

**FIGURE 1:**
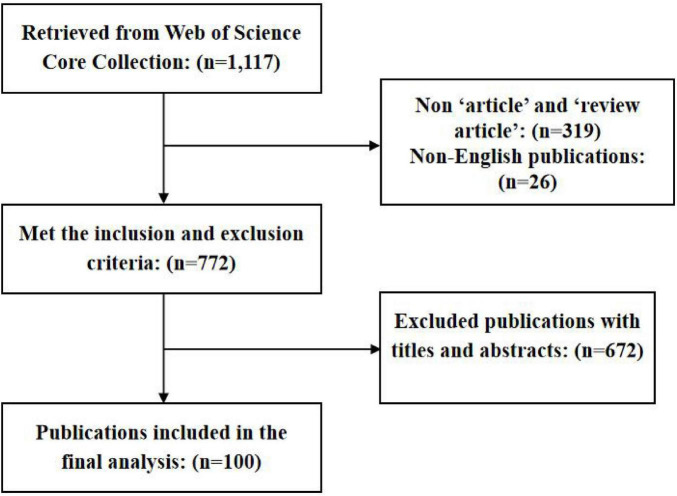
Flow chart.

In terms of exclusion criteria, we excluded precluded non-article, non-review, and non-English publications. Two authors read the titles and abstracts of each article independently to ensure their eligibility from the original set of 100 most cited publications. Any disagreements were resolved through consensus or by involving a senior author. Figure 1 illustrates the flow chart of the document selection procedure.

### 2.3 Data analysis and visualization

Microsoft Excel was used to perform descriptive analysis on data collected from the Web of Science Core Collection on authors, countries, journals, institutions, and literature. JCR Science Edition 2022 was used to determine the journal impact factor. VOS viewer is a bibliometric networks analysis program written in Java that can process thousands of scientific literature articles and produce unimodal undirected networks of publication patterns. Using VOS viewer, this study performed a co-authorship analysis, which showed collaboration between authors and countries, and a co-occurrence analysis of keywords, revealing the research focus and topic frontier. In VOS viewer, we conducted co-authorship analysis and visualized collaboration networks among authors, countries, and institutions. Co-authorship analysis is based on the frequency of two items (e.g., author, country, institution) appearing together in the same paper. In the collaboration network, each node represents an item (e.g., author, country, institution), and the node’s size is determined by the number of publications involving that item—the more publications, the larger the node. In VOS viewer, nodes appear if they have been involved in at least five articles. The thickness of the lines between nodes indicates the frequency of co-occurrence in articles; thicker lines represent more frequent collaboration. Nodes within the same collaboration cluster are defined using the same color.

## 3 Results

According to our analysis, 1,117 scientific journals have been published on research on aerobic fitness in children and adolescents. The 100 most cited articles’ characteristics and the number of citations are shown in Supplementary Table 1. The number of total citations ranged from 51 to 419 (mean 104.1 ± 69.08; median: 77.5). The article titled “*A neuroimaging investigation of the association between aerobic fitness, hippocampal volume, and memory performance in preadolescent children*” was ranked at the top with 419 citations. This study explores these links in 9- and 10-year-old children using MRI scans, finding that higher-fit children showed larger hippocampal volumes and better relational memory, mediated by fitness levels (30). The second one was “*Independent associations of physical activity and cardiorespiratory fitness with metabolic risk factors in children: the European youth heart study*,” which revealed that PA and cardiorespiratory fitness (CRF) independently link to individual and clustered metabolic risk factors in children (31), and with 407 citations. The top three is titled “*Aerobic fitness and neurocognitive function in healthy preadolescent children*,” which argues that fitness is positively related to neuroelectric attention and working memory indexes and response speed in children (32). However, the oldest paper in the list, titled “A Comparison of the pwc170 and 20-most tests of Aerobic Fitness in Adolescent Schoolchildren,” was published in 1990 and was ranked 22 with only 129 citations (33).

We identified 18 productive journals with at least two articles each (Table 1). The British Journal of Sports Medicine (*n* = 9) and Medicine and Science in Sports and Exercise, which were in JCR’s Q1 (top 25% of journals in the field) of the sports sciences category, are the two core journals with the highest yields. This finding highlights the high quality of research papers on aerobic fitness in children and adolescents published in the JCR evaluation system.

**TABLE 1:** Number of most-cited articles by journals.

Rank	Journal	Papers	Number of citations	CPP	JIF2022	JCI2022
1	British Journal of Sports Medicine	9	1,060	117.8	18.6	4.26
= 2	European Journal of Applied Physiology	4	328	82.0	3.0	0.94
= 2	Medicine and Science in Sports and Exercise	4	594	148.5	4.1	1.81
= 2	Pediatric Exercise Science	4	402	100.5	1.8	0.83
= 5	European Journal of Cardiovascular Prevention and Rehabilitation	3	422	140.7	3.691*	NA
= 5	International Journal of Obesity	3	355	118.3	4.9	1.08
= 5	Journal of Pediatrics	3	221	73.7	5.1	1.78
= 5	Pediatrics	3	293	97.7	8.0	3.02
= 9	European Heart Journal	2	180	90.0	39.3	6.79
= 9	European Journal of Clinical Nutrition	2	108	54.0	4.7	0.81
= 9	European Journal of Pediatrics	2	148	74.0	3.6	1.38
= 9	International Journal of Environmental Research and Public Health	2	115	57.5	4.614**	0.93**
= 9	International Journal of Pediatric Obesity	2	188	94.0	3.025*	NA
= 9	Journal of Applied Physiology	2	380	190.0	3.3	1.12
= 9	Mental Health and Physical Activity	2	152	76.0	4.7	0.96
= 9	Obesity	2	189	94.5	6.9	1.43
= 9	PLoS One	2	159	79.5	3.7	0.91
= 9	Sports Medicine	2	226	113.0	9.8	2.11

*The last impact factor of this journal was obtained in 2013;

**The last impact factor and JIF of this journal was obtained in 2021.

**FIGURE 2:**
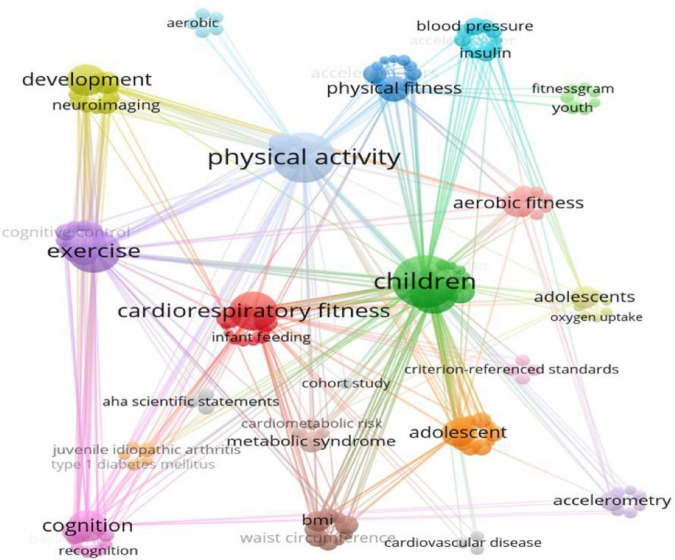
Keyword co-occurrence analysis.

The contribution to retrieved articles was made by 29 countries (Table 2). Both developed and developing countries were in the top ten most productive countries, with the United States at the top. Most contributors are from developed and Western countries, indicating a high level of concern and research intensity on aerobic fitness in children and adolescents in these countries. Based on the co-occurrence keywords (Figure 2), six clusters of all the included terms represent the research focus and achievements over the past years. Three research hotspots stand out. First, most of the terms in the red cluster were from neuroscience (e.g., hippocampus and its related brain functions) and developmental psychology (e.g., academic achievement) in the context of aerobic health in children and adolescents. Two other prominent research studies focus on obesity and cardiovascular disease and their related risk factors.

**TABLE 2:** Number of top-cited articles by country.

Rank	Country^a^	Papers	Number of citations	CPP
1	USA	45	5,881	130.7
2	Sweden	18	1,620	90.0
= 3	England	14	1,633	116.6
= 3	Spain	14	1,387	99.1
5	Norway	12	1,490	124.2
= 6	Australia	9	991	110.1
= 6	Canada	9	782	86.9
8	Denmark	7	858	122.6
9	Portugal	6	958	159.7
10	Netherlands	5	552	110.4

^a^Countries with at least two articles were summarized.

**FIGURE 3:**
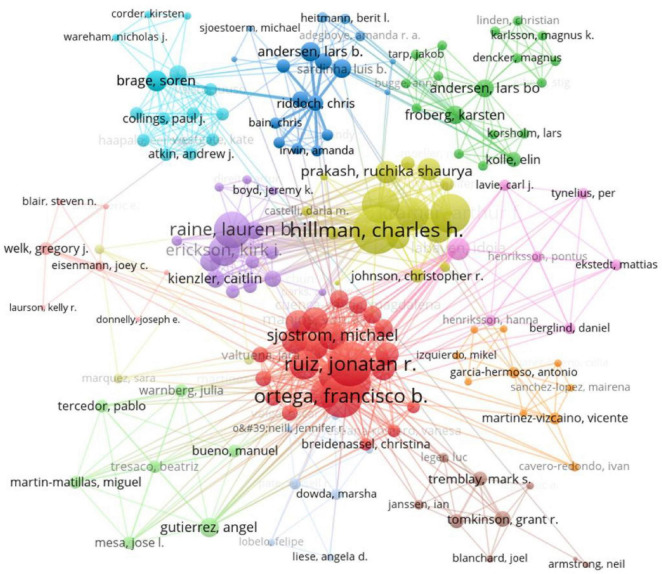
Authors cooperation network mapping.

A publication co-authorship network of different authors was generated (Figure 3). Through the co-authorship analysis, we found more than 6 clusters containing hundreds of authors. Among them, three high-producing research teams with principal authors who published the most articles are Hillman, Charles H. (*n* = 18), Ruiz, Jonatan R. (*n* = 10), and Raine, Lauren B. (*n* = 8).

## 4 Discussion

This study analyzed the 100 most cited publications on aerobic fitness in children and adolescents and presented their bibliometric findings. In total, 520 papers were regarded as the 500 most cited physical activity papers. The citation analysis applied in this study provides quantitative information about authors, journals, countries, and institutions by identifying highly cited studies on aerobic fitness in children and adolescents as well as the development of the field over time (34). The most cited papers on aerobic fitness in children and adolescents were cited 47 to 403 times. This is lower when compared with citations for publications on the broad field such as physical activity [range = 297–8,068; (27)], hypertension [range = 582–7,248; (35)] and diabetes [range = 964–17,779; (36)] as well as specific research fields such as the research on football [range = 251–869; (37)] and clinical orthopedic sports medicine [range = 229–1,629; (38)]. However, this is not surprising given that the citation pattern may differ across many specialties and depends on several factors, such as the scope and spread of the field, the number of researchers in a field, the publishing model of the majority of journals in the field, geographic origin and primary language of researchers in the field, and the number of papers considered as the top cited in the field (27, 34, 39, 40).

This study found that 6% of the most cited papers on aerobic fitness in children and adolescents were published before 2000, and all were published in the 1990s. However, papers published after 2000 received more citations than the older ones. This may be because older publications may lose recognition due to the availability of more recent citation alternative papers due to advancement in the field (27). For example, an old theoretical framework is replaced by a new framework or an old measurement technique is replaced by a more accurate one. Further, cross-citation or inappropriate citation practices involving the omission of citing a source may also be one reason for this phenomenon (41). This was evidenced by the oldest article titled “A Comparison of the pwc170 and 20-most tests of aerobic fitness in Adolescent schoolchildren,” published in 1990, ranked 22 with only 129 citations. The study compared two aerobic fitness tests in adolescent schoolchildren. Conversely, the top-ranked study titled “*Independent associations of physical activity and cardiorespiratory fitness with metabolic risk factors in children: the European youth heart study*” examined the associations of physical activity and cardiorespiratory fitness with metabolic risk factors in a European study on children. While the exact reason for this is unclear, one potential explanation for this could be that the combination of factors such as the nature of the study (i.e., associations), its broader focus (i.e., covering physical activity, cardiorespiratory fitness, and metabolic risk factors), author group [i.e., Ekelund et al. (31)], and publishing journal (i.e., Diabetology; ∼ 10 impact factor) resulted in higher citations.

Most of the most cited publications on aerobic fitness in children and adolescents were published by authors affiliated with institutions from developed countries (e.g., USA, England, Norway, Sweden, and Canada), with very few published by authors from developing countries (e.g., China). These findings are supported by bibliometric studies on the 100 most cited papers in many health fields (27, 34, 36, 40). This may be because of several reasons. First, developed countries have comparatively more researchers, more robust research networks, and higher research funding than developing countries (27, 34, 40). Second, famous researchers or active researchers in the field usually work with a specific group of individuals based in other developed countries. For example, Ekelund U had the strongest research links with researchers from other developed countries. Third, it has been argued that authors from developed countries are likely to cite authors’ work from the same country (42). Such trends have been observed in previous publications on physical activity (27, 41). Finally, as developed countries have a better economic ranking, infrastructure (e.g., research labs), and research support (e.g., funding), they are likely to have a higher quantity and quality of biomedical publications (41, 43).

The most cited papers on aerobic fitness in children and adolescents were published in diverse medical journals. The top 10 prolific journals included journals such as the British Journal of Sports Medicine (*n* = 9; CPP: 105.0) and Medicine and Science in Sports and Exercise (*n* = 6; CPP: 137.2). The list included journals specific to sports science, physiology, endocrinology and metabolism, pediatrics, and multidisciplinary journals. It may be noted that the tendency for highly cited papers to get published in general or specialized journals varies across different fields (27, 34). Although we did not determine whether papers published in open-access journals are cited more compared to traditional journals, we could yet infer that traditional journals are still dominant in publishing papers on aerobic fitness in children and adolescents because the most prolific journals, including the British Journal of Sports Medicine, Medicine and Science in Sports and Exercise, European Journal of Applied Physiology, and European Journal of Cardiovascular Prevention and Rehabilitation, among others. However, future research is needed to determine the effect of the publishing model on citations in different fields, particularly in sports and exercise.

The top 100 most cited papers on aerobic fitness in children and adolescents’ papers are focused on fields of neuroscience (e.g., hippocampus and its related brain functions), developmental psychology (e.g., academic achievement), obesity, and cardiovascular disease. It shows that most landmark papers on aerobic fitness in children and adolescents focus more on cognition and memory than health outcomes. It might be because most health outcomes are examined in adults or across the lifespan. Therefore, studies published on children and adults might have been missed.

Since 2000, research into aerobic fitness among children and adolescents has surged due to escalating concerns about childhood obesity and related health issues stemming from sedentary lifestyles (44, 45). Developed countries lead in this area, leveraging advanced research infrastructure and funding, which enable sophisticated studies using technologies like MRI and EEG to explore the impact of fitness on physical and cognitive health (30, 46). The prioritization of cognition and health research reflects a growing body of evidence demonstrating that aerobic fitness enhances cognitive function, attention, memory, and overall mental wellbeing in youth (6, 47, 48). This recognition has prompted policymakers and educators to advocate for integrating physical activity into school curricula to improve physical health and academic outcomes among young people (6, 49–52).

In addition to the factors above, sleep plays a pivotal role in influencing children and adolescents’ physical and mental health (53–55). Sufficient duration and sleep quality are crucial for maintaining aerobic fitness and promoting overall brain health. It can be argued that several lifestyle factors, including physical activity, nutrition, and sleep, are increasingly recognized for their profound effects on brain health and aerobic fitness. Past studies indicate that regular physical activity improves cardiovascular health and enhances cognitive function and overall brain health through increased neurogenesis and synaptic plasticity (56, 57). Similarly, balanced nutrition, including adequate intake of essential nutrients like omega-3 fatty acids and antioxidants, supports cognitive processes and may influence aerobic fitness by optimizing energy metabolism and muscle function (58, 59). Furthermore, quality sleep plays a crucial role in memory consolidation, emotional regulation, and physical recovery, all vital for maintaining optimal aerobic fitness levels (60). Understanding the interplay among these factors and their cumulative impact on children and teenagers’ health is imperative for devising effective strategies to foster their growth (61, 62).

To promote physical activity and healthy lifestyles among children and youth, concerted efforts from families, schools, and the government are essential, emphasizing positive feedback. The government should address issues like outdated educational structures and inadequate sports facilities to provide optimal conditions for teenagers. Schools can support this by designing tailored sports programs that inspire enthusiasm and autonomy among students, offering choices and reinforcing competence through positive feedback. Group activities foster a sense of belonging while integrating music, which enhances cognitive health benefits. Parents should actively guide their children, promptly engaging their interests to meet their physical needs effectively. Overall, improving physical education for children and youth requires creating supportive macro conditions and prioritizing individual student needs to enhance their sports participation.

Some limitations of this study should be acknowledged. First, using the Web of Science Core Collection database allows us to find papers published back to 1950, but it does not cover documents from Medline or Scopus databases. Second, there might be some inaccuracies in the bibliometric parameters (e.g., differences in authors’ names or affiliations) included in the Web of Science Core Collection. Third, the keywords used in the search for the most cited papers may also have affected the study’s findings. Finally, the number of citations yielded from the Web of Science Core Collection may differ from other databases used for bibliometric analyses (e.g., Scopus). Although the results may be comparable to different databases, the difference in the coverage of databases suggests that the list of the 100 most cited papers on aerobic fitness in children and adolescents presented in this study may not be generalisable to other databases. Nonetheless, this is the first bibliometric study on the 100 most cited publications on aerobic fitness in children and adolescents. In addition, compared to other bibliographic databases, the Web of Science database has been suggested to be methodologically better for bibliometric studies of top-cited articles because it allows us to find papers published back to 1950.

Future research on aerobic fitness in children and adolescents should focus on several key areas. (1) Standardized Testing: Standardized and consistent fitness testing methodologies are needed for better comparisons across studies and countries; (2) Comprehensive Interventions: Future research should focus on interventions that can effectively increase physical activity and improve fitness levels among children and adolescents; (3) Broader Geographic Scope: Including more data from underrepresented regions can provide a more global perspective on fitness trends; (4) Longitudinal Studies: Conducting long-term studies can help understand the impact of early-life fitness on adult health outcomes and physical and cognitive development; (5) Investigating the genetic factors and lifestyle choices: Influencing fitness can help create personalized programs. Research should also explore the impact of different types of physical activities and the socio-environmental factors that affect activity levels. Finally, incorporating modern technology, like wearable fitness trackers, can enhance data collection and promote sustained physical activity. These directions will inform public health policies and educational programs to improve children and youth health and wellbeing.

## 5 Conclusion

This bibliometric analysis offers synthesized findings based on the 100 most-cited articles, providing information on quantitative information about authors, journals, countries, and institutions. This study highlights that the most cited papers on aerobic fitness in children and adolescents were published in diverse medical-related journals and the most recognized aerobic fitness research in children and adolescents was published after 2000. In the field of aerobic fitness in children and adolescents, most authors were affiliated with institutions from developed countries and highly cited research rooted from the neuroscience field. This study also offers a basis for future research and practice. Future research contributions to the field from authors and institutions from developing countries are needed.

## Data Availability

The original contributions presented in this study are included in the article/Supplementary material, further inquiries can be directed to the corresponding authors.
