# Supplementary material for: Research on aerobic fitness in children and adolescents: a bibliometric analysis based on the 100 most-cited articles
Source: Front Med (Lausanne). 2024 Sep 25;11:1409532. doi: 10.3389/fmed.2024.1409532 (PMC11461214; doi:10.3389/fmed.2024.1409532)
Supplement: Supplementary file 1 [file Table_1.docx]

**Supplementary Table 1. List of included papers**

| **Rank** | **Authors** | **Title** | **Journal** | **Country** | | **Total citations** | |
| --- | --- | --- | --- | --- | --- | --- | --- |
| 1 | Chaddock, L.et al (2010) | A neuroimaging investigation of the association between aerobic fitness, hippocampal volume, and memory performance in preadolescent children | Brain Research | USA | | 419 | |
| 2 | Ekelund, U.et al (2007) | Independent associations of physical activity and cardiorespiratory fitness with metabolic risk factors in children: the European youth heart study | Diabetologia | England | | 407 | |
| 3 | Hillman, CH.et al (2005) | Aerobic fitness and neurocognitive function in healthy preadolescent children | Medicine and Science in Sports and Exercise | USA | | 314 | |
| 4 | Hillman, CH.et al (2009) | Aerobic Fitness and Cognitive Development: Event-Related Brain Potential and Task Performance Indices of Executive Control in Preadolescent Children | Developmental Psychology | USA | | 298 | |
| 5 | Chaddock, L.et al (2010) | Basal Ganglia Volume Is Associated with Aerobic Fitness in Preadolescent Children | Developmental Neuroscience | USA | | 238 | |
| 6 | Pontifex, MB.et al (2011) | Cardiorespiratory Fitness and the Flexible Modulation of Cognitive Control in Preadolescent Children | Journal of Cognitive Neuroscience | USA | | 231 | |
| 7 | Anderssen, SA.et al (2007) | Low cardiorespiratory fitness is a strong predictor for clustering of cardiovascular disease risk factors in children independent of country, age and sex | European Journal of Cardiovascular Prevention & Rehabilitation | Norway | | 226 | |
| 8 | Chaddock, L.et al (2011) | A Review of the Relation of Aerobic Fitness and Physical Activity to Brain Structure and Function in Children | Journal of the International Neuropsychological Society | USA | | 215 | |
| 9 | Raghuveer, G.et al (2020) | Cardiorespiratory Fitness in Youth: An Important Marker of Health: A Scientific Statement From the American Heart Association | Circulation | USA | | 207 | |
| 10 | Rowlands, AV.et al (1999) | Relationship between activity levels, aerobic fitness, and body fat in 8- to 10-yr-old children | Journal of Applied Physiology | England | | 203 | |
| =11 | Ruiz, JR.et al (2016) | Cardiorespiratory fitness cut points to avoid cardiovascular disease risk in children and adolescents; what level of fitness should raise a red flag? A systematic review and meta-analysis | British Journal of Sports Medicine | Spain | | 202 | |
| =11 | Khan, NA.et al (2014) | The Relation of Childhood Physical Activity and Aerobic Fitness to Brain Function and Cognition: A Review | Pediatric Exercise Science | USA | | 202 | |
| 13 | Buck, SM.et al (2008) | The relation of aerobic fitness to stroop task performance in preadolescent children | Medicine and Science in Sports and Exercise | USA | | 196 | |
| 14 | Tomkinson, GR.et al (2019) | Temporal trends in the cardiorespiratory fitness of children and adolescents representing 19 high-income and upper middle-income countries between 1981 and 2014 | British Journal of Sports Medicine | USA | | 193 | |
| 15 | Artero, EG.et al (2011) | Muscular and cardiorespiratory fitness are independently associated with metabolic risk in adolescents: the HELENA study | Pediatric Diabetes | Spain | | 188 | |
| 16 | Steele, RM.et al (2008) | Physical activity, cardiorespiratory fitness, and the metabolic syndrome in youth | Journal of Applied Physiology | England | | 177 | |
| 17 | Pate, RR.et al (2006) | Cardiorespiratory fitness levels among US youth 12 to 19 years of age - Findings from the 1999-2002 National Health and Nutrition Examination Survey | Archives of Pediatrics & Adolescent Medicine | USA | | 174 | |
| =18 | Mintjens, S.et al (2018) | Cardiorespiratory Fitness in Childhood and Adolescence Affects Future Cardiovascular Risk Factors: A Systematic Review of Longitudinal Studies | Sports Medicine | Netherlands | | 158 | |
| =18 | Armstrong, N.et al (2011) | Aerobic fitness and its relationship to sport, exercise training and habitual physical activity during youth | British Journal of Sports Medicine | England | | 158 | |
| 20 | Burgi, F.et al (2011) | Relationship of physical activity with motor skills, aerobic fitness and body fat in preschool children: a cross-sectional and longitudinal study (Ballabeina) | International Journal of Obesity | Switzerland | | 155 | |
| 21 | Voss, MW.et al (2011) | Aerobic fitness is associated with greater efficiency of the network underlying cognitive control in preadolescent children | Neuroscience | USA | | 139 | |
| 22 | Chaddock, L.et al (2012) | Childhood aerobic fitness predicts cognitive performance one year later | Journal of Sports Sciences | USA | | 135 | |
| =23 | Chaddock, L.et al (2012) | A functional MRI investigation of the association between childhood aerobic fitness and neurocognitive control | Biological Psychology | USA | | 133 | |
| =23 | Ortega, FB.et al (2007) | Cardiorespiratory fitness and sedentary activities are associated with adiposity in adolescents | Obesity | Spain | | 133 | |
| =25 | Ortega, FB.et al (2007) | Cardiorespiratory fitness and sedentary activities are associated with adiposity in adolescents | Obesity | Spain | | 129 | |
| =25 | Boreham, CAG.et al (1990) | A comparison of the pwc170 and 20-mst tests of aerobic fitness in adolescent schoolchildren | Journal of Sports Medicine and Physical Fitness | Northern Ireland | | 129 | |
| =27 | Haapala, EA.et al (2013) | Cardiorespiratory Fitness and Motor Skills in Relation to Cognition and Academic Performance in Children - A Review | Journal of Human Kinetics | Finland | | 128 | |
| =27 | Chaddock, L.et al (2011) | Aerobic Fitness and Executive Control of Relational Memory in Preadolescent Children | Medicine and Science In Sports and Exercise | USA | | 128 | |
| =27 | Hurtig-Wennlof, A.et al (2007) | Cardiorespiratory fitness relates more strongly than physical activity to cardiovascular disease risk factors in healthy children and adolescents: the European Youth Heart Study | European Journal of Cardiovascular Prevention & Rehabilitation | Sweden | | 128 | |
| =30 | Hogstrom, G.et al (2014) | High aerobic fitness in late adolescence is associated with a reduced risk of myocardial infarction later in life: a nationwide cohort study in men | European Heart Journal | Sweden | | 117 | |
| =30 | Benson, AC.et al (2006) | Muscular strength and cardiorespiratory fitness is associated with higher insulin sensitivity in children and adolescents | International Journal of Pediatric Obesity | Australia | | 117 | |
| 32 | Eisenmann, JC.et al (2005) | Aerobic fitness, body mass index, and CVD risk factors among adolescents: the Quebec family study | International Journal of Obesity | USA | | 115 | |
| 33 | Marques, A.et al (2018) | How does academic achievement relate to cardiorespiratory fitness, self-reported physical activity and objectively reported physical activity: a systematic review in children and adolescents aged 6-18 years | British Journal of Sports Medicine | Portugal | | 114 | |
| 34 | Dencker, M.et al (2006) | Daily physical activity and its relation to aerobic fitness in children aged 8-11 years | European Journal of Applied Physiology | Sweden | | 113 | |
| 35 | Wong, PCH.et al (2008) | Effects of a 12-week exercise training programme on aerobic fitness, body composition, blood lipids and C-reactive protein in adolescents with obesity | Annals Academy of Medicine Singapore | Singapore 107 | | |  |
| 36 | DuBose, KD.et al (2007) | Aerobic fitness attenuates the metabolic syndrome score in normal-weight, at-risk-for-overweight, and overweight children | Pediatrics | USA | | 106 | |
| =37 | Eisenmann, JC.et al (2007) | Aerobic fitness, fatness and the metabolic syndrome in children and adolescents | Acta Paediatrica | USA | | 104 | |
| =37 | Hussey, J.et al (2007) | Relationship between the intensity of physical activity, inactivity, cardiorespiratory fitness and body composition in 7-10-year-old Dublin children | British Journal of Sports Medicine | Ireland | | 104 | |
| 39 | Armstrong, N.et al (1996) | Is peak VO2 a maximal index of children's aerobic fitness? | International Journal of Sports Medicine | England | | 102 | |
| 40 | Meckel, Y.et al (2009) | Relationship among repeated sprint tests, Aerobic fitness, And anaerobic fitness in elite adolescent soccer players | Journal of Strength and Conditioning Research | Israel | | 98 | |
| 41 | Roberts, CK.et al (2010) | Low Aerobic Fitness and Obesity Are Associated with Lower Standardized Test Scores in Children | Journal of Pediatrics | USA | | 93 | |
| =42 | Chaddock-Heyman, L.et al (2014) | The importance of physical activity and aerobic fitness for cognitive control and memory in children | Monographs of The Society for Research In Child Development | USA | | 92 | |
| =42 | Herting, MM.et al (2012) | Aerobic fitness relates to learning on a virtual Morris Water Task and hippocampal volume in adolescents | Behavioural Brain Research | USA | | 92 | |
| 44 | Garcia-Hermoso, A.et al (2020) | Association of Cardiorespiratory Fitness Levels During Youth With Health Risk Later in Life: A Systematic Review and Meta-analysis | Jama Pediatrics | Spain | | 91 | |
| 45 | Lobelo, F.et al (2009) | Validity of Cardiorespiratory Fitness Criterion-Referenced Standards for Adolescents | Medicine and Science In Sports and Exercise | USA | | 90 | |
| 46 | Moliner-Urdiales, D.et al (2011) | Associations of muscular and cardiorespiratory fitness with total and central body fat in adolescents: The HELENA Study | British Journal of Sports Medicine | Spain | | 89 | |
| 47 | Maggio, ABR.et al (2010) | Reduced physical activity level and cardiorespiratory fitness in children with chronic diseases | European Journal of Pediatrics | Switzerland | | 88 | |
| =48 | Hogstrom, G.et al (2016) | Aerobic fitness in late adolescence and the risk of early death: a prospective cohort study of 1.3 million Swedish men | International Journal of Epidemiology | Sweden | | 87 | |
| =49 | Eisenmann, JC.et al (2011) | Aerobic Fitness Percentiles for US Adolescents | American Journal of Preventive Medicine | USA | | 87 | |
| 50 | Stratton, G.et al (2007) | Cardiorespiratory fitness and body mass index of 9-11-year-old English children: a serial cross-sectional study from 1998 to 2004 | International Journal of Obesity | England | | 85 | |
| 51 | de Oliveira, RG.et al (2016) | Physical Activity, Sedentary Behavior, Cardiorespiratory Fitness and Metabolic Syndrome in Adolescents: Systematic Review and Meta-Analysis of Observational Evidence | Plos One | Brazil | | 84 | |
| =52 | Aadland, KN.et al (2017) | Relationships between physical activity, sedentary time, aerobic fitness, motor skills and executive function and academic performance in children | Mental Health and Physical Activity | Norway | 82 |  |  |
| =52 | Knopfli, BH.et al (2008) | Effects of a multidisciplinary inpatient intervention on body composition, aerobic fitness, and quality of life in severely obese girls and boys | Journal of Adolescent Health | Switzerland | | 82 | |
| =52 | Cairney, J.et al (2007) | Developmental coordination disorder and cardiorespiratory fitness in children | Pediatric Exercise Science | Canada | | 82 | |
| 55 | Lang, JJ.et al (2018) | Making a Case for Cardiorespiratory Fitness Surveillance Among Children and Youth | Exercise and Sport Sciences Reviews | Canada | | 81 | |
| 56 | Ruiz, JR.et al (2015) | Cardiorespiratory fitness and ideal cardiovascular health in European adolescents | Heart | Spain | | 79 | |
| 57 | Aires, L.et al (2010) | Intensity of Physical Activity, Cardiorespiratory Fitness, and Body Mass Index in Youth | Journal of Physical Activity & Health | Portugal | | 76 | |
| 58 | Chaddock-Heyman, L.et al (2015) | The Role of Aerobic Fitness in Cortical Thickness and Mathematics Achievement in Preadolescent Children | Plos One | USA | | 75 | |
| =59 | Kristensen, PL.et al (2010) | The association between aerobic fitness and physical activity in children and adolescents: the European youth heart study | European Journal of Applied Physiology | Norway | | 74 | |
| =59 | Ball, GDC.et al (2004) | Insulin sensitivity, cardiorespiratory fitness, and physical activity in overweight hispanic youth | Obesity Research | USA | | 74 | |
| =59 | Rowland, TW.et al (1991) | Effects of obesity on aerobic fitness in adolescent females | American Journal of Diseases of Children | USA 74 | |  |  |
| =62 | Eisenmann, JC.et al (2007) | Combined influence of cardiorespiratory fitness and body mass index on cardiovascular disease risk factors among 8-18 year old youth: The Aerobics Center Longitudinal Study | International Journal of Pediatric Obesity | USA | | 71 | |
| =62 | Ekelund, U.et al (2001) | Physical activity in relation to aerobic fitness and body fat in 14-to 15-year-old boys and girls | European Journal of Applied Physiology | Sweden | | 71 | |
| =64 | Lambourne, K.et al (2013) | Indirect and direct relations between aerobic fitness, physical activity, and academic achievement in elementary school students | Mental Health and Physical Activity | USA | | 70 | |
| =64 | Dencker, M.et al (2007) | Gender differences and determinants of aerobic fitness in children aged 8-11 years | European Journal of Applied Physiology | Sweden | | 70 | |
| =64 | Klasson-Heggebo, L.et al (2006) | Graded associations between cardiorespiratory fitness, fatness, and blood pressure in children and adolescents | British Journal of Sports Medicine | Norway | | 70 | |
| =64 | ARMSTRONG, N.et al (1995) | Aerobic fitness of prepubescent children | Annals of Human Biology | England | | 70 | |
| 68 | Schaefer, L.et al (2014) | Outdoor Time Is Associated with Physical Activity, Sedentary Time, and Cardiorespiratory Fitness in Youth | Journal of Pediatrics | Canada | | 69 | |
| =69 | Collings, PJ.et al (2017) | Cross-Sectional Associations of Objectively-Measured Physical Activity and Sedentary Time with Body Composition and Cardiorespiratory Fitness in Mid-Childhood: The PANIC Study | Sports Medicine | England | | 68 | |
| =69 | Reed, KE.et al (2005) | Arterial compliance in young children: the role of aerobic fitness | European Journal of Cardiovascular Prevention & Rehabilitation | Canada | | 68 | |
| 71 | Perez, M.et al (2014) | Aerobic Fitness Is Associated With Lower Risk of Hospitalization in Children With Cystic Fibrosis | Pediatric Pulmonology | Spain | | 67 | |
| =72 | Wittberg, RA.et al (2012) | Children's Aerobic Fitness and Academic Achievement: A Longitudinal Examination of Students During Their Fifth and Seventh Grade Years | American Journal of Public Health | USA | | 66 | |
| =72 | Monti, JM.et al (2012) | Aerobic fitness enhances relational memory in preadolescent children: The FITKids randomized control trial | Hippocampus | USA | | 66 | |
| =74 | Pozuelo-Carrascosa, DP.et al (2018) | Effectiveness of school-based physical activity programmes on cardiorespiratory fitness in children: a meta-analysis of randomised controlled trials | British Journal of Sports Medicine | Spain | | 65 | |
| =74 | Adegboye, ARA.et al (2011) | Recommended aerobic fitness level for metabolic health in children and adolescents: a study of diagnostic accuracy | British Journal of Sports Medicine | Norway | | 65 | |
| 76 | Takken, T.et al (2002) | Aerobic fitness in children with juvenile idiopathic arthritis: A systematic review | Journal of Rheumatology | Netherlands | | 64 | |
| =77 | Henriksson, H.et al (2020) | Cardiorespiratory fitness, muscular strength, and obesity in adolescence and later chronic disability due to cardiovascular disease: a cohort study of 1 million men | EUROPEAN HEART JOURNAL | Sweden 63 | |  | |
| =77 | Cao, M.et al (2019) | Effect of High-Intensity Interval Training versus Moderate-Intensity Continuous Training on Cardiorespiratory Fitness in Children and Adolescents: A Meta-Analysis | International Journal of Environmental Research and Public Health | China | | 63 | |
| =77 | Wu, CT.et al (2011) | Aerobic Fitness and Response Variability in Preadolescent Children Performing a Cognitive Control Task | Neuropsychology | USA | | 63 | |
| =80 | Kao, SC.et al (2017) | Muscular and Aerobic Fitness, Working Memory, and Academic Achievement in Children | Medicine and Science In Sports and Exercise | USA | | 62 | |
| =80 | Stewart KJ.et al (1976) | EFFECTS OF PHYSICAL-TRAINING ON CARDIORESPIRATORY FITNESS IN CHILDREN | RESEARCH QUARTERLY | England | | 62 | |
| =82 | Chaddock-Heyman, L.et al (2016) | Aerobic fitness is associated with greater hippocampal cerebral blood flow in children | Developmental Cognitive Neuroscience | USA | | 61 | |
| =82 | Scudder, MR.et al (2014) | The association between aerobic fitness and language processing in children: Implications for academic achievement | Brain and Cognition | USA | | 61 | |
| =84 | Tomkinson GR.et al (2019) | The 20-m Shuttle Run: Assessment and Interpretation of Data in Relation to Youth Aerobic Fitness and Health | PEDIATRIC EXERCISE SCIENCE | Australia | | 60 | |
| =84 | Bailey, DP.et al (2012) | Associations between cardiorespiratory fitness, physical activity and clustered cardiometabolic risk in children and adolescents: the HAPPY study | European Journal of Pediatrics | England | | 60 | |
| 86 | Huang, T.et al (2015) | Associations of Adiposity and Aerobic Fitness with Executive Function and Math Performance in Danish Adolescents | Journal of Pediatrics | Norway | | 59 | |
| =87 | Weisman J.et al (2019) | Interpreting Aerobic Fitness in Youth: The Fallacy of Ratio Scaling | PEDIATRIC EXERCISE SCIENCE | England | | 58 | |
| =87 | Senechal, M.et al (2013) | Cardiorespiratory Fitness and Adiposity in Metabolically Healthy Overweight and Obese Youth | Pediatrics | Canada | | 58 | |
| =89 | Martinez-Gomez, D.et al (2011) | Excessive sedentary time and low cardiorespiratory fitness in European adolescents: the HELENA study | Archives of Disease in Childhood | Spain | | 57 | |
| =89 | He, QQ.et al (2011) | Physical activity, cardiorespiratory fitness, and obesity among Chinese children | Preventive Medicine | China | | 57 | |
| =91 | Braam, KI.et al (2016) | Cardiorespiratory fitness and physical activity in children with cancer | SUPPORTIVE CARE IN CANCER | Netherland | | 56 | |
| =91 | McGavock, JM.et al (2009) | Cardiorespiratory Fitness and the Risk of Overweight in Youth: The Healthy Hearts Longitudinal Study of Cardiometabolic Health | Obesity | Canada | | 56 | |
| =91 | Nassis, GP.et al (2005) | Central and total adiposity are lower in overweight and obese children with high cardiorespiratory fitness | European Journal of Clinical Nutrition | Greece | | 56 | |
| 94 | Fitzpatrick, JF.et al (2018) | Dose-Response Relationship Between Training Load and Changes in Aerobic Fitness in Professional Youth Soccer Players | INTERNATIONAL JOURNAL OF SPORTS PHYSIOLOGY AND PERFORMANCE | England | | 55 | |
| =95 | Padilla-Moledo, C.et al (2012) | Positive health, cardiorespiratory fitness and fatness in children and adolescents | European Journal of Public Health | Spain | | 54 | |
| =95 | Dencker, M.et al (2008) | Daily physical activity related to aerobic fitness and body fat in an urban sample of children | Scandinavian Journal of Medicine & Science in Sports | Sweden | | 54 | |
| =95 | Lawlor, DA.et al (2008) | Associations of birth size and duration of breast feeding with cardiorespiratory fitness in childhood: findings from the Avon Longitudinal Study of Parents and Children (ALSPAC) | European Journal of Epidemiology | England | | 54 | |
| =98 | Martin-Smith, R.et al (2020) | High Intensity Interval Training (HIIT) Improves Cardiorespiratory Fitness (CRF) in Healthy, Overweight and Obese Adolescents: A Systematic Review and Meta-Analysis of Controlled Studies | INTERNATIONAL JOURNAL OF ENVIRONMENTAL RESEARCH AND PUBLIC HEALTH | England | | 52 | |
| =98 | Lee, SJ.et al (2007) | Cardiorespiratory fitness and abdominal adiposity in youth | European Journal of Clinical Nutrition | USA | | 52 | |
| 100 | Lindgren, M.et al (2017) | Cardiorespiratory fitness and muscle strength in late adolescence and long-term risk of early heart failure in Swedish men | EUROPEAN JOURNAL OF PREVENTIVE CARDIOLOGY | Sweden | | 51 | |
